# [Retracted] Long non-coding RNA CCAT2 promotes prostate cancer cell proliferation and invasion by regulating the Wnt/β-catenin signaling pathway

**DOI:** 10.3892/ol.2026.15703

**Published:** 2026-06-15

**Authors:** Peng He, Guoguang Xiong, Wei Guo, Guanjun Jiang, Yun Li, Hao Li

Oncol Lett 20: 97, 2020; DOI: 10.3892/ol.2020.11958

Subsequently to the publication of the above article, a concerned reader drew to the Editor's attention that, regarding the Transwell invasion assay data shown in Fig. 5I on p. 7 for the PC3 cell line (the lower row of panels), the ‘siNC’, ‘LiCL’ and ‘siCCAT2+LiCL’ data panels contained overlapping sections, such that this trio of panels were apparently derived from the same original source, where different experimental conditions were reported in the figure. In addition, in Fig. 3H, the protein bands shown for the TCF7L2 row of data with the DU145 cell line for the ‘si-NC’ and ‘si-CCAT2+miR-217 inhibitor’ experiments (the first and the second lanes in the gel) appeared to be strikingly similar, suggesting that this figure may have potentially undergone inappropriate editing.

After having evaluated these data independently in the Editorial Office, the concerns of the reader were shown to have been well-founded. Therefore, in view of the uncertainties regarding the assembly of the above mentioned western blot data in Fig. 3H, the Editor of *Oncology Letters* has decided that this paper should be retracted from the Journal on account of a lack of confidence in the presented data. The authors were asked for an explanation to account for these concerns, but the Editorial Office did not receive a reply. The Editor apologizes to the readership for any inconvenience caused.

